# Screening high-risk individuals for primary gastric carcinoma: evaluating overall survival probability score in the presence and absence of lymphatic metastasis post-gastrectomy

**DOI:** 10.1186/s12957-024-03481-8

**Published:** 2024-07-25

**Authors:** Wenqing Qu, Ling Li, Jinfeng Ma, Yifan Li

**Affiliations:** 1 Hepatobiliary, Pancreatic and Gastrointestinal Surgery, Shanxi Hospital Affiliated to Carcinoma Hospital, Chinese Academy of Medical Sciences, Shanxi Province Carcinoma Hospital, Carcinoma Hospital Affiliated to Shanxi Medical University, Taiyuan, 030013 Shanxi P.R. China; 2 Shanxi Medical University, 030013 Taiyuan, Shanxi P.R. China

**Keywords:** Gastric cancer, Lymphatic metastasis, Overall survival, Nomogram, Risk stratification

## Abstract

**Objective:**

The aim of this study was to develop and validate prognostic models for predicting overall survival in individuals with gastric carcinoma, specifically focusing on both negative and positive lymphatic metastasis.

**Methods:**

A total of 1650 patients who underwent radical gastric surgery at Shanxi Cancer Hospital between May 2002 and December 2020 were included in the analysis. Multiple Cox Proportional Hazards analysis was performed to identify key variables associated with overall survival in both negative and positive lymphatic metastasis cases. Internal validation was conducted using bootstrapping to assess the prediction accuracy of the models. Calibration curves were used to demonstrate the accuracy and consistency of the predictions. The discriminative abilities of the prognostic models were evaluated and compared with the 8th edition of AJCC-TNM staging using Harrell’s Concordance index, decision curve analysis, and time-dependent receiver operating characteristic curves.

**Results:**

The nomogram for node-negative lymphatic metastasis included variables such as age, pT stage, and maximum tumor diameter. The C-index for this model in internal validation was 0.719, indicating better performance compared to the AJCC 8th edition TNM staging. The nomogram for node-positive lymphatic metastasis included variables such as gender, age, maximum tumor diameter, neural invasion, Lauren classification, and expression of Her-2, CK7, and CD56. The C-index for this model was 0.674, also outperforming the AJCC 8th edition TNM staging. Calibration curves, time-dependent receiver operating characteristic curves, and decision curve analysis for both nomograms demonstrated excellent prediction ability. Furthermore, significant differences in prognosis between low- and high-risk groups supported the models’ strong risk stratification performance.

**Conclusion:**

This study provides valuable risk stratification models for lymphatic metastasis in gastric carcinoma, encompassing both node-positive and negative cases. These models can help identify low-risk individuals who may not require further intervention, while high-risk individuals can benefit from targeted therapies aimed at addressing lymphatic metastasis.

## Introduction

Globally, gastric cancer remains a formidable force, ranking as the fifth most prevalent cancer and the fourth leading cause of mortality [1]. With advancements in medical technologies, various effective treatments such as endoscopy, surgical procedures, radiotherapy, chemotherapy, and immunotherapy have greatly improved the outlook for individuals diagnosed with gastric cancer [2–6]. To achieve the best possible outcomes in terms of both cure and survival, a radical resection stands out as the most productive approach, rendering it the preferred treatment option for resettable, non-metastatic gastric cancer [7, 8]. The depth of tumor invasion and the involvement of lymph nodes, both of which are carefully assessed in almost all staging systems for gastric cancer, holds immense significance as independent prognostic factors for overall survival (OS) following a successful microscopic margin-negative (R0) resection [9–11].

In our retrospective study, we discovered that the average overall survival (OS) for patients without lymphatic metastasis was 44.1 ± 24.05 months, while for those with lymphatic metastasis, it was 37.35 ± 21.64 months. Similarly, patients without lymphatic metastasis had an average progression-free survival (PFS) of 41.73 ± 23.83 months, while those with lymphatic metastasis had a PFS of 31.17 ± 23.83 months. These findings emphasize the significance of considering lymphatic metastasis when predicting the prognosis of gastric carcinoma. The primary objective of this study was to develop precise nomograms capable of accurately predicting the outcome of patients with gastric carcinoma, specifically focusing on those with and without lymphatic metastasis. Additionally, we utilized risk stratification to differentiate between patients who would benefit from appropriate chemotherapy, those who should avoid excessive treatment, and those who could potentially benefit from combined treatments. By incorporating the principles of precision medicine, these nomograms and risk stratification will provide exact guidance for postoperative treatment of gastric carcinoma. Ultimately, this approach will enable personalized treatment for individual patients, improving treatment outcomes.

## Method

### Data collection

For the research, data from 1650 individuals who had undergone radical gastric surgery for the treatment of gastric carcinoma at Shanxi Cancer Hospital between May 2002 and December 2020 were examined. Among these individuals, 557 gastric carcinoma patients without lymphatic metastasis were selected and randomly divided into two groups in a ratio of 7:3. The first group consisted of 386 cases and served as the training cohort, while the second group consisted of 171 cases and served as the validation cohort. Similarly, a total of 1093 gastric carcinoma patients with lymphatic metastasis were selected and randomly divided into two groups in a ratio of 7:3. The training cohort comprised 778 cases, while the validation cohort comprised 315 cases.

**Fig. 1:**
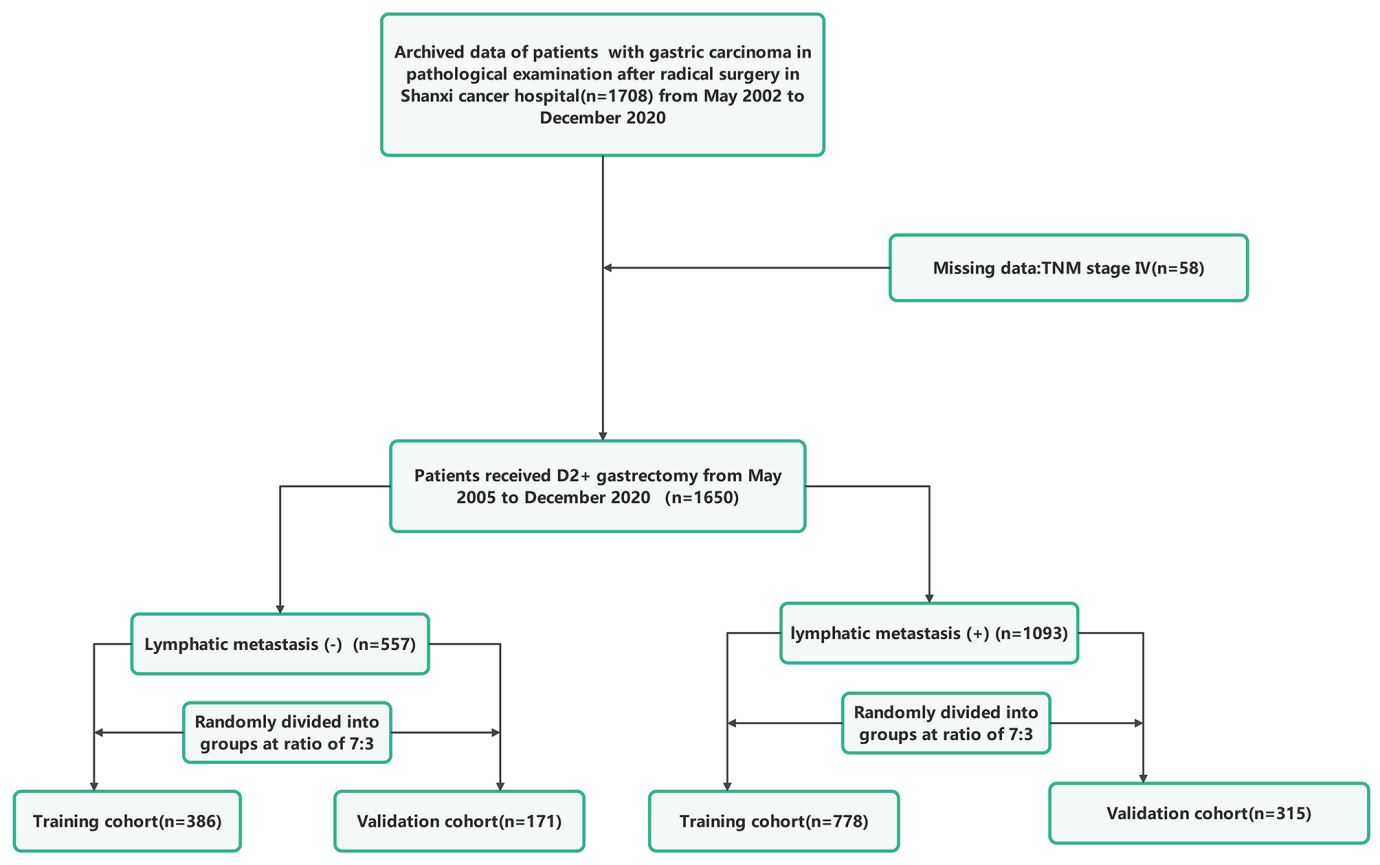
The flowchart illustrating the enrollment process of the study population in both the training and validation cohorts of gastric cancer

Inclusion criteria for the study required patients to have histological confirmation of gastric adenocarcinoma, complete clinicopathological and follow-up data, no severe organ damage post-surgery, and no other unrelated malignant tumors or causes of death. Patients with systemic tumors, incomplete clinical data, palliative or bypass surgery, or non-gastric cancer were excluded. Tumor staging was based on the AJCC 8th TNM classification. The study protocol was approved by the Ethics Committee of Shanxi Cancer Hospital and ethical principles were followed and informed consent has been obtained from all patients involved, indicating that they have given voluntary and informed agreement to participate in the study. Patient data were anonymized and kept confidential. Figure 1 outlines the research process in a flowchart.

In order to be included in the study, patients had to meet specific criteria. These criteria consisted of a confirmed diagnosis of gastric cancer through histological examination and undergoing surgery with the aim of curing the disease (R0-R1). The researchers analyzed various factors that influenced the outcome of the surgery, such as gender, age at the time of surgery, presence of vascular and neural invasion, tumor stage (pT stage), number of positive lymph nodes, cancer stage (TNM stage according to the American Joint Committee’s 8th edition), Lauren classification, maximum tumor diameter, type of gastrectomy performed, presence of omentum metastasis, surgical margin status, degree of complications based on the Clavien-Dindo classification, expression of specific biomarkers (AE1/AE3, Ki67, CK20, CDX-2, SATB-2, SYN, CGA, CD56, MLH1, PMS2, Her-2, MSH2, and MSH6), and overall survival (OS). The follow-up time was determined using electronic medical records of hospital visits and communication with the oncologist. The follow-up period began with the last hospital visit and ended with the last contact with the surgeon. OS was calculated as the time between surgery and death. This retrospective research study utilized clinical data that had been collected and complied with institutional guidelines and regulations. All participants provided informed consent, following the principles outlined in the Declaration of Helsinki [12, 13].

### Statistical analysis

Descriptive statistics were utilized to summarize the data, featuring categorical variables presented as absolute numerical counts and continuous variables represented by the medians of the interquartile range. Kaplan-Meier curves were employed to visually illustrate OS. The association between OS and various factors was examined through multivariate Cox regression analysis, with results reported as hazard ratios along with corresponding 95% confidence intervals (CI) and *p* values. Statistical significance was defined as a *p* value less than 0.05. Data processing was conducted using a variety of software packages, including R software 4.3.2(The University of Auckland, Auckland, New Zealand) and SPSS 25.0( IBM Corp., Armonk, NY, USA).

### Nomogram performance

The development of these nomograms involved conducting Cox regression analysis on several OS-related parameters, with internal validation being assessed through 10,000 iterations, and external validation via a separate validation cohort. Risk factors from the stepwise model were categorized using clinical benchmarks or tertiles, which facilitated the creation of the prediabetes score model. These risk factors, treated as categorical variables, were input into a stepwise Cox proportional hazards model to derive a novel β coefficient. The scoring system was then established by multiplying regression coefficients by three and rounding to the nearest integer to determine the weights. This scoring system was implemented in a user-friendly questionnaire format for primary care providers. The total score was divided into two risk categories: low and high risk. Furthermore, we evaluated the efficacy of our risk score model for predicting OS in patients with gastric cancer, including those with lymphatic metastasis. Survival probabilities and time-to-event variables were computed using the Kaplan-Meier method. The log-rank test was employed to compare OS probabilities between the low and high risk groups (quartiles of risk score). Model performance was assessed via calibration and discrimination tests, which included Harrell’s concordance index (C-Index) and the area under the receiver operating characteristic curve (AUC). An AUC value ranging from 0.5 to 0.7 signifies poor discrimination, while values from 0.7 to 0.9 indicate moderate performance; an AUC above 0.9 reflects excellent performance. Calibration curves were utilized to evaluate the consistency of the results, and decision curve analysis (DCA) was conducted to gauge the clinical utility of the nomograms.

## Results

### Basic characteristics of training cohort of lymphatic metastasis (-) and lymphatic metastasis (+)

In the training cohort, we included 386 gastric cancer patients with negative lymphatic metastasis and positive lymphatic metastasis including 778 gastric cancer patients, each variable was balanced in the two groups (Table 1).

**Table 1:** Demographics of study population

Variables	Lymphatic metastasis (-) (*n* = 386) Training cohort	Lymphatic metastasis (+) (*n* = 778) Training cohort
	Mean ± SD/No(%)	Mean ± SD/No(%)
Gender		
Male	313(81.1%)	609(78.3%)
Female	73(18.9%)	169(21.7%)
Age (years)	58.61 ± 9.88	58.68 ± 10.107
pT stage		
T1	191(49.5%)	25(3.2%)
T2	34(8.8%)	21(2.7%)
T3	93(24.1%)	260(33.4%)
T4	68(17.6%)	472(60.7%)
pTNM stage		
I	226(58.5%)	16(2.1%)
II	158(40.9%)	128(16.5%)
III	2(0.5%)	634(81.5%)
Vascular invasion		
Negative	314(81.3%)	234(30.1%)
Positive	72(18.7%)	544(69.9%)
Neural invasion		
Negative	313(81.1%)	311(40%)
Positive	73(18.9%)	467(60%)
Lauren classification		
Intestinal	274(71.0%)	196(25.2%)
Diffuse	47(12.2%)	354(45.5%)
Mixed	65(16.8%)	228(29.3%)
Type of gastrectomy		
Proximal	76(19.7%)	54(6.9%)
Distal	156(40.4%)	231(29.7%)
Total	152(39.4%)	493(63.4%)
Omentum metastasis		
Negative	385(99.7%)	749(96.3%)
Positive	1(0.3%)	29(3.7%)
Surgical margin		
Negative	377(97.7%)	730(93.8%)
Positive	9(2.3%)	48(6.2%)
Her-2		
Negative	254(65.8%)	475(61.1%)
Positive	132(34.2%)	303(38.9%)
AE1/AE3		
Negative	187(48.4%)	63(8.1%)
Positive	199(51.6%)	715(91.9%)
Ki167	33 ± 25.839	66.65 ± 19.184
CK7		
Negative	198(51.3%)	387(49.7%)
Positive	188(48.7%)	391(50.3%)
CK20		
Negative	277(71.8%)	571(73.4%)
Positive	109(28.2%)	207(26.6%)
CDX-2		
Negative	257(66.6%)	393(50.5%)
Positive	129(33.4%)	385(49.5%)
SATB-2		
Negative	337(87.3%)	610(78.4%)
Positive	49(12.7%)	168(21.6%)
SYN		
Negative	296(76.7%)	560(72.0%)
Positive	90(23.3%)	218(28.0%)
CGA		
Negative	299(77.5%)	669(86.0%)
Positive	87(22.5%)	109(14.0%)
CD56		
Negative	279(72.3%)	468(60.2%)
Positive	107(27.7%)	310(39.8%)
MLH1		
Negative	99(25.6%)	50(6.4%)
Positive	287(74.4%)	728(93.6%)
PMS2		
Negative	180(46.6%)	144(18.5%)
Positive	206(53.4%)	634(81.5%)
MSH2		
Negative	100(25.9%)	54(6.9%)
Positive	286(74.1%)	724(93.1%)
MSH6		
Negative	106(27.5%)	52(6.7%)
Positive	280(72.5%)	726(93.3%)
Maximum diameter of Tumor	3.634 ± 2.282	5.609 ± 2.459
Tumor location		
upper	187(48.4%)	397(51.0%)
middle	60(15.5%)	134(17.2%)
lower	136(35.2%)	243(31.2%)
multiple	3(0.8%)	4(0.5%)

Abbreviations: SD: standard deviation; No: number

### Development and validation of the prediction model of OS for negative lymphatic metastasis

**Fig. 2:**
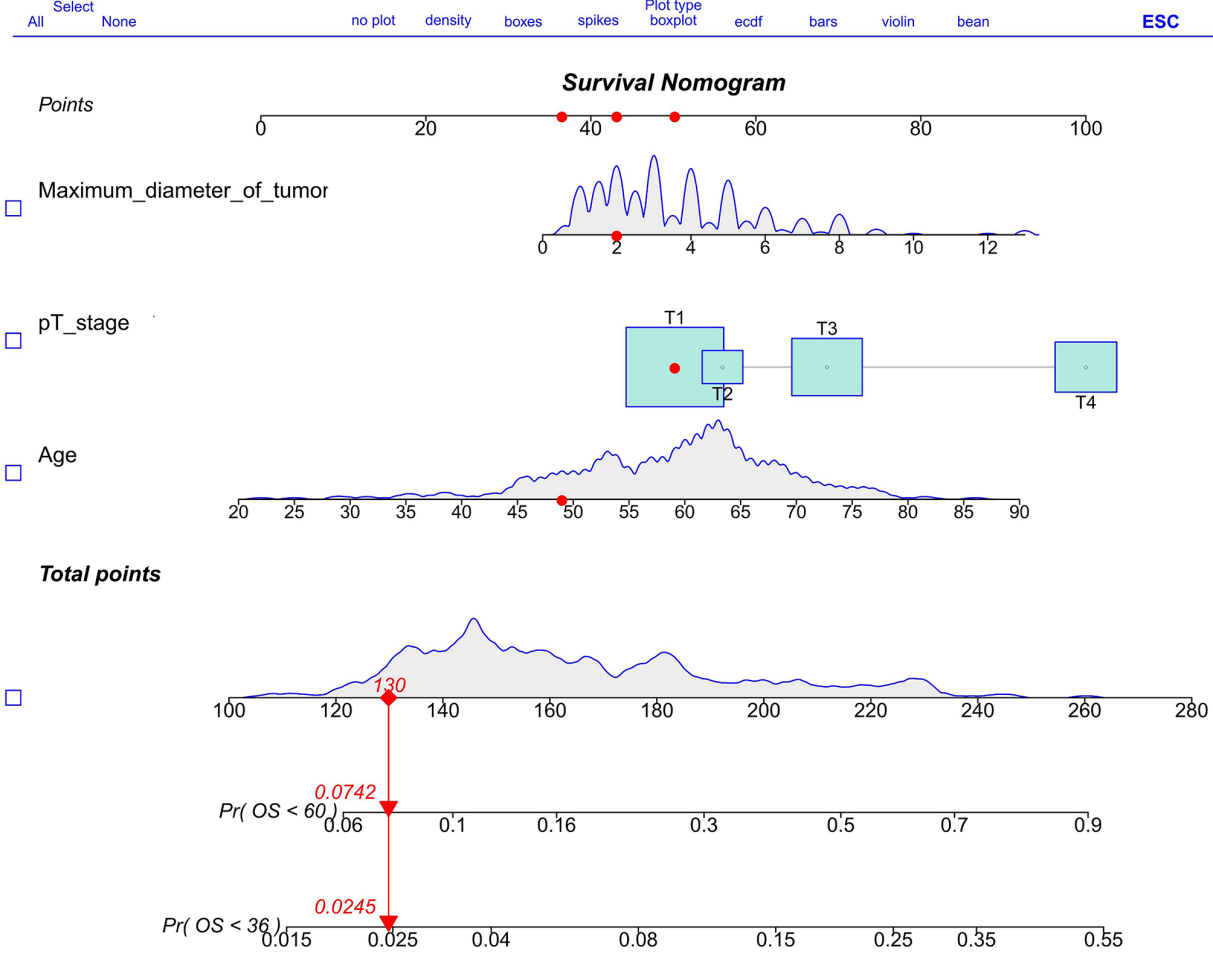
Nomogram model designed to predict the 3-year and 5-year OS of patients with negative lymphatic metastasis

**Fig. 3:**
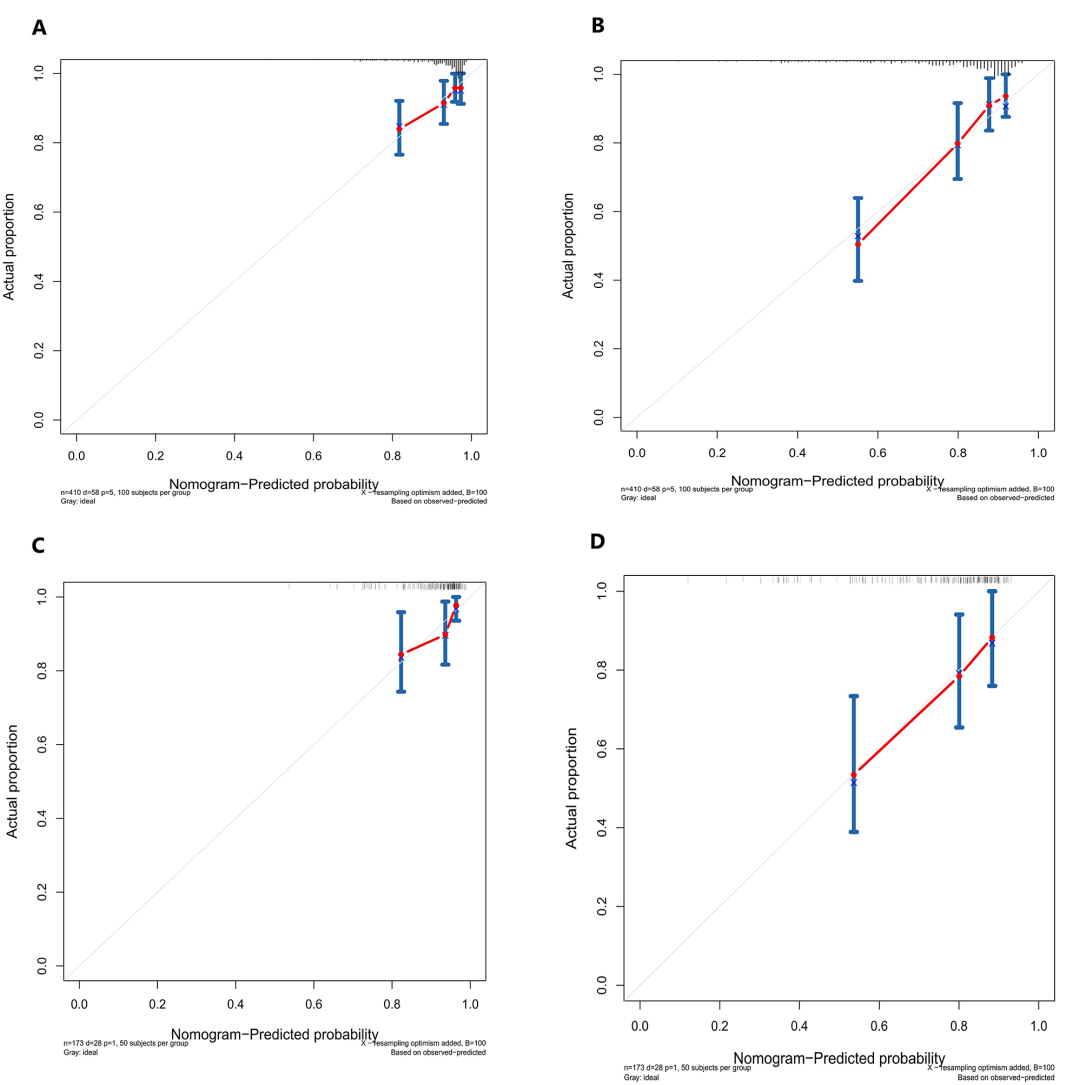
Calibration curves demonstrating the accuracy of the internal and external validation in predicting the 3-year and 5-year OS of patients with negative lymphatic metastasis. **A**. Internal validation of 3-year OS. **B**. External validation of 3-year OS. **C**. Internal validation of 5-year OS. **D**. External validation of 5-year OS

A multivariate Cox regression analysis was conducted to identify independent prognostic factors for OS in patients without lymphatic metastasis. Significant factors including age, pT stage, and maximum tumor diameter were determined from the results presented in Table 2, which were obtained from a training cohort of 386 gastric cancer patients. By integrating these variables into a nomogram model for lymphatic metastasis (-), we were able to predict the 3-year and 5-year OS probabilities for these patients as shown in Fig. 2. Based on the nomogram, each variable was assigned a specific point, and the points were summed to determine the probability of OS onset at 3 and 5 years. This nomogram model takes into account various factors known to impact favorable outcomes and offers a reliable forecast of a patient’s 3-year and 5-year OS. Figure 3 illustrates the nomogram model’s ability to predict a positive outcome for gastric cancer patients by considering the factors influencing 3-year and 5-year OS. In the training cohort, the C-index for the node-negative predictor (-) was calculated to be 0.719 (95%CI: 0.653–0.786), indicating a relatively dependable predictive ability. Furthermore, in comparison to the discrimination of the AJCC 8th edition TNM staging, the nomogram demonstrated superior performance with a C-index of 0.658 (95%CI: 0.586–0.731).

**Table 2:** Multivariate analysis of OS of training cohort of negative lymphatic metastasis and analyzed by Cox regression

	B	SE	Wald	df	*p*	Exp(B)	EXP(95%CI)
age	0.034	0.016	4.756	1	0.029	1.035	1.003–1.068
pT stage			13.344	3	0.004		1.211–2.359
T1 Vs. T2	0.150	0.641	0.054	1	0.816	1.161	0.330–4.081
T1 Vs. T3	0.473	0.424	1.247	1	0.264	1.606	0.699–3.686
T1 Vs. T4	1.281	0.380	11.345	1	0.001	3.600	1.708–7.587
Maximum diameter of Tumor	0.118	0.059	4.026	1	0.045	1.125	1.003–1.263

Abbreviations: B: regression coefficient; SE: standard error; *df*: degree of freedom; HR: hazard ratio; CI: confidence interval

**Fig. 4:**
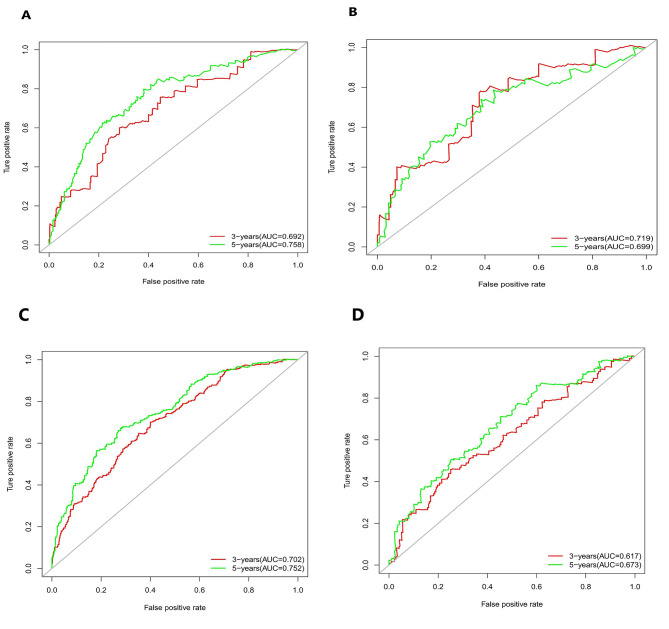
Time-dependent receiver operating characteristic (t-ROC) curves showcasing the performance of the internal and external validation in predicting the OS of both negative and positive lymphatic metastasis. (**A**) Internal validation of negative lymphatic metastasis. (**B**) External validation of negative lymphatic metastasis (**C**) Internal validation of positive lymphatic metastasis. (**D**) External validation of positive lymphatic metastasis

**Fig. 5:**
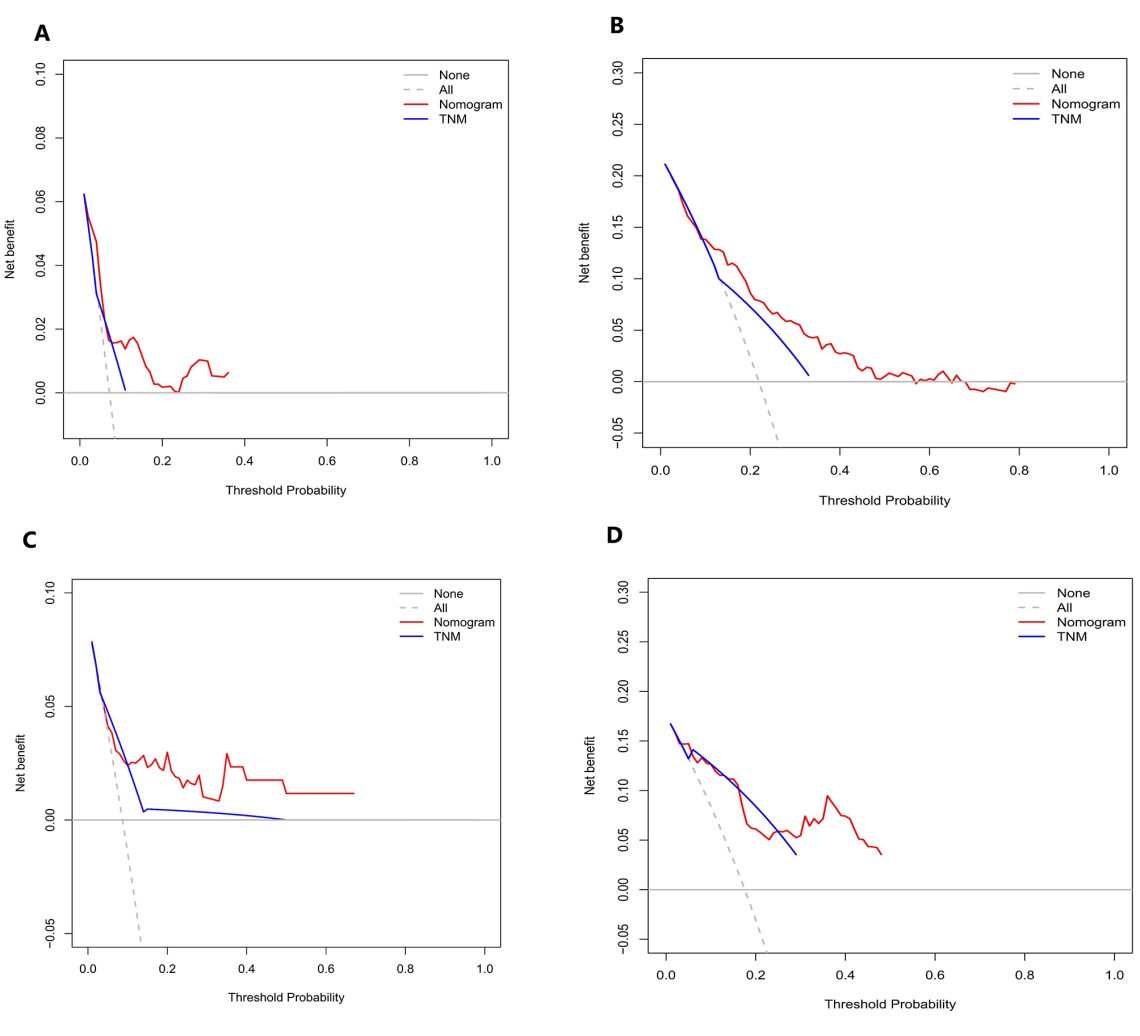
Decision curve analysis (DCA) results of the internal and external validation to predict the 3-year and 5-year OS of patients with negative lymphatic metastasis. **A**. Internal validation of 3-year OS. **B**. External validation of 3-year OS. **C**. Internal validation of 5-year OS. **D**. External validation of 5-year OS

The calibration curves in Fig. 3A, B and C, and 3D illustrate the effectiveness of the nomogram in predicting OS at 3 and 5 years. The alignment between predicted and observed outcomes in both internal and external validation demonstrates the accuracy of the nomogram. The nomogram’s ability to distinguish between different outcomes is further supported by the time-dependent ROC analysis in internal validation. The AUC values for 3-year and 5-year OS are 0.692 (95%CI: 0.564–0.780) and 0.758 (95%CI: 0.702–0.852), respectively. External validation also shows promising results with AUC values of 0.719 (95%CI: 0.503–0.830) for 3-year OS and 0.699 (95%CI : 0.591–0.858) for 5-year OS (Fig. 4A and B). To assess the clinical utility of the nomogram, decision analysis curve (DCA) was utilized to compare the predictions of 5-year and 3-year OS between the nomogram and the AJCC 8th edition TNM staging. The internal validation C-index for the nomogram was 0.719 (95%CI: 0.653–0.786), higher than the C-index of 0.683 (95%CI: 0.658–0.731) for the AJCC 8th edition TNM staging. Similarly, the external validation C-index for the nomogram was 0.715 (95%CI: 0.614–0.816), exceeding the C-index of 0.697 (95%CI: 0.653–0.742) for the AJCC 8th edition TNM staging. The higher C-index values for the nomogram model in both internal and external validation indicate its superior predictive ability compared to the AJCC 8th edition TNM staging (Fig. 5A, B, C and D).

### Risk scoring of stratification system of OS for negative lymphatic metastasis

**Fig. 6:**
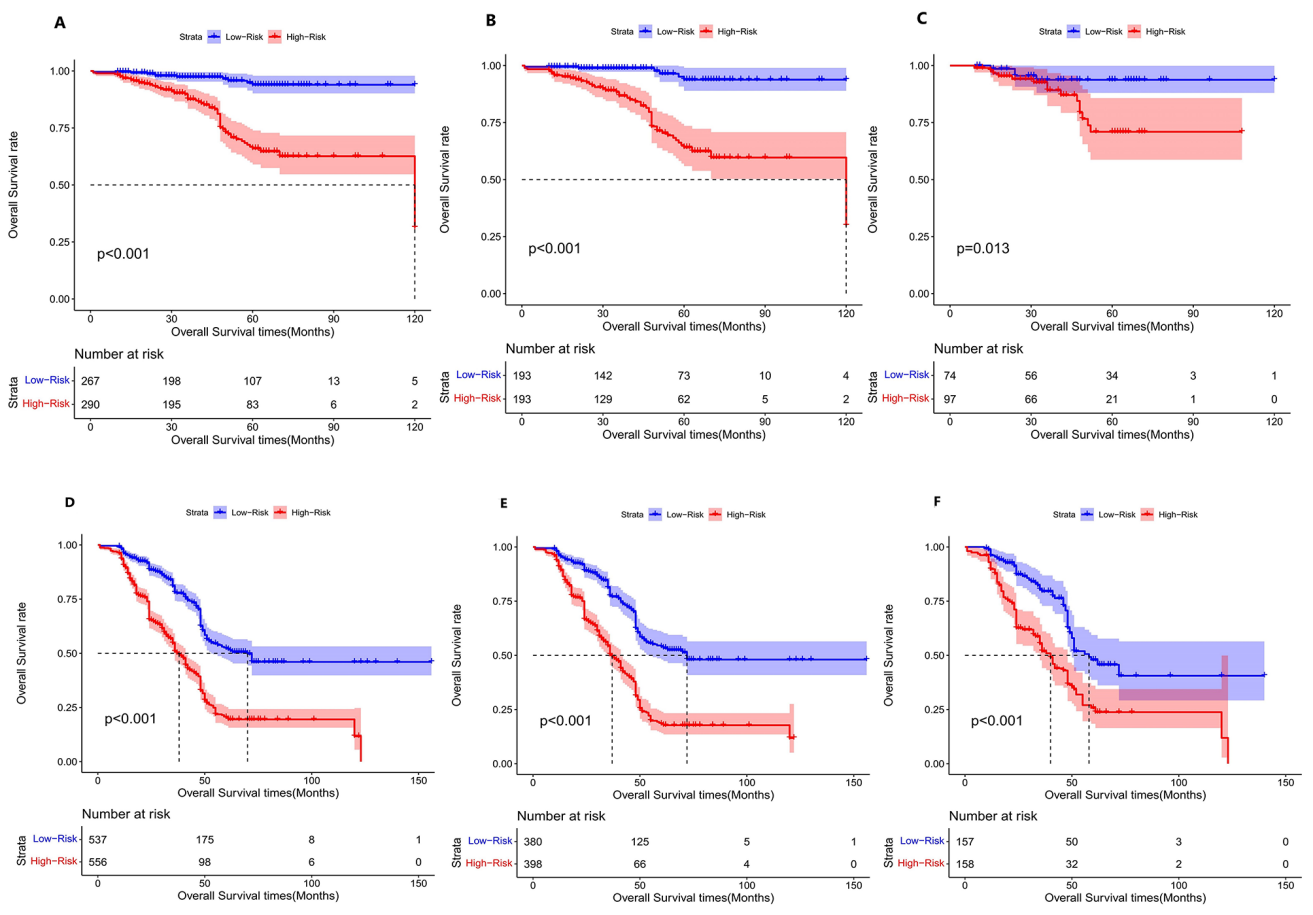
Kaplan-Meier survival curves depicting the survival outcomes of patients with different scores of negative and positive lymphatic metastases in the overall cohort, training cohort, and validation cohort. (**A**) all cohort of negative lymphatic metastasis (**B**) training cohort of negative lymphatic metastasis (**C**) validation cohort of negative lymphatic metastasis (**D**) all cohort of positive lymphatic metastasis (**E**) training cohort of positive lymphatic metastasis (**F**) validation cohort of positive lymphatic metastasis

**Fig. 7:**
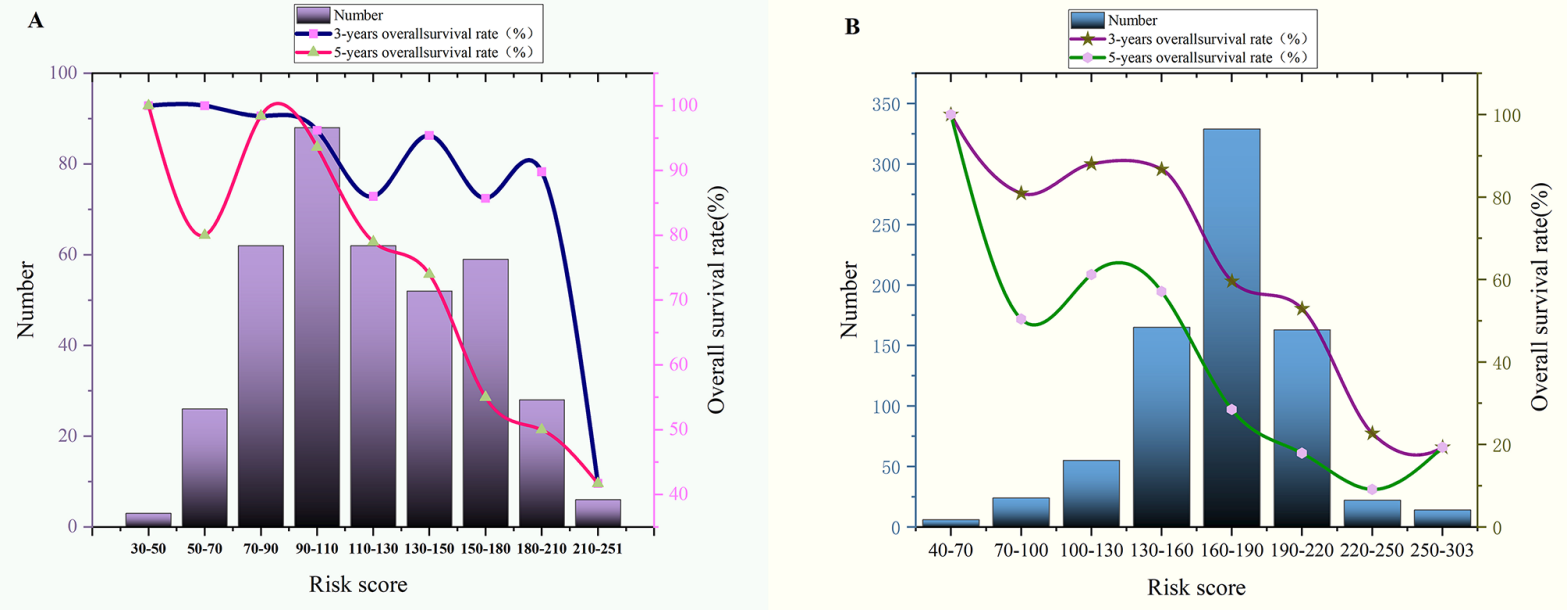
The correlation between the risk score and the 3-year and 5-year OS rate of patients (**A**) Negative lymphatic metastasis. (**B**) Positive lymphatic metastasis

Based on the final lymphatic metastasis nomogram model, each patient was assigned a score and placed into a category. The X-tile software was utilized to determine the cutoff value for OS scores in the training group, which consisted of 386 patients. Subsequently, the log-rank test was employed to compare survival times among different risk groups. The prognostic nomogram was utilized to calculate total scores. Utilizing a cutoff value of 109.05, the entire cohort of 557 individuals was divided into two distinct groups with varying mortality risks, as depicted in Fig. 6. The low-risk group (score ≤ 109.05) comprised 193 patients from the training group (*n* = 386) and 74 patients from the validation group (*n* = 171). Conversely, the high-risk group (score > 109.05) comprised 193 patients from the training group (*n* = 386) and 105 patients from the validation group (*n* = 171). Figure 6 showcases the OS curves for the overall and training groups, demonstrating highly significant *p* values of less than 0.001, along with a *p* value of 0.013 in the validation group. It is worth noting that the median OS for the entire cohort, encompassing both low and high-risk groups, has not been reached. The notable disparities in prognosis between the two risk groups further validate the exceptional performance of our model in risk stratification. Figure 7A illustrates the association between risk score and overall survival rate. There is a downward trend in the 5-year overall survival rate as the risk score increases, particularly when the score exceeds 90 (90–110: 93.6%, 180–210: 50%, 210–251: 41.7%). Similarly, the 3-year overall survival rate experiences a significant decrease when the risk score surpasses 180 (180–210: 89.8%, 210–251: 41.7%).

These findings offer a concise visual representation of the relationship between risk scores and survival rates, aligning seamlessly with the risk stratification OS system.

### Development and validation of the prediction model of OS for positive lymphatic metastasis

**Fig. 8:**
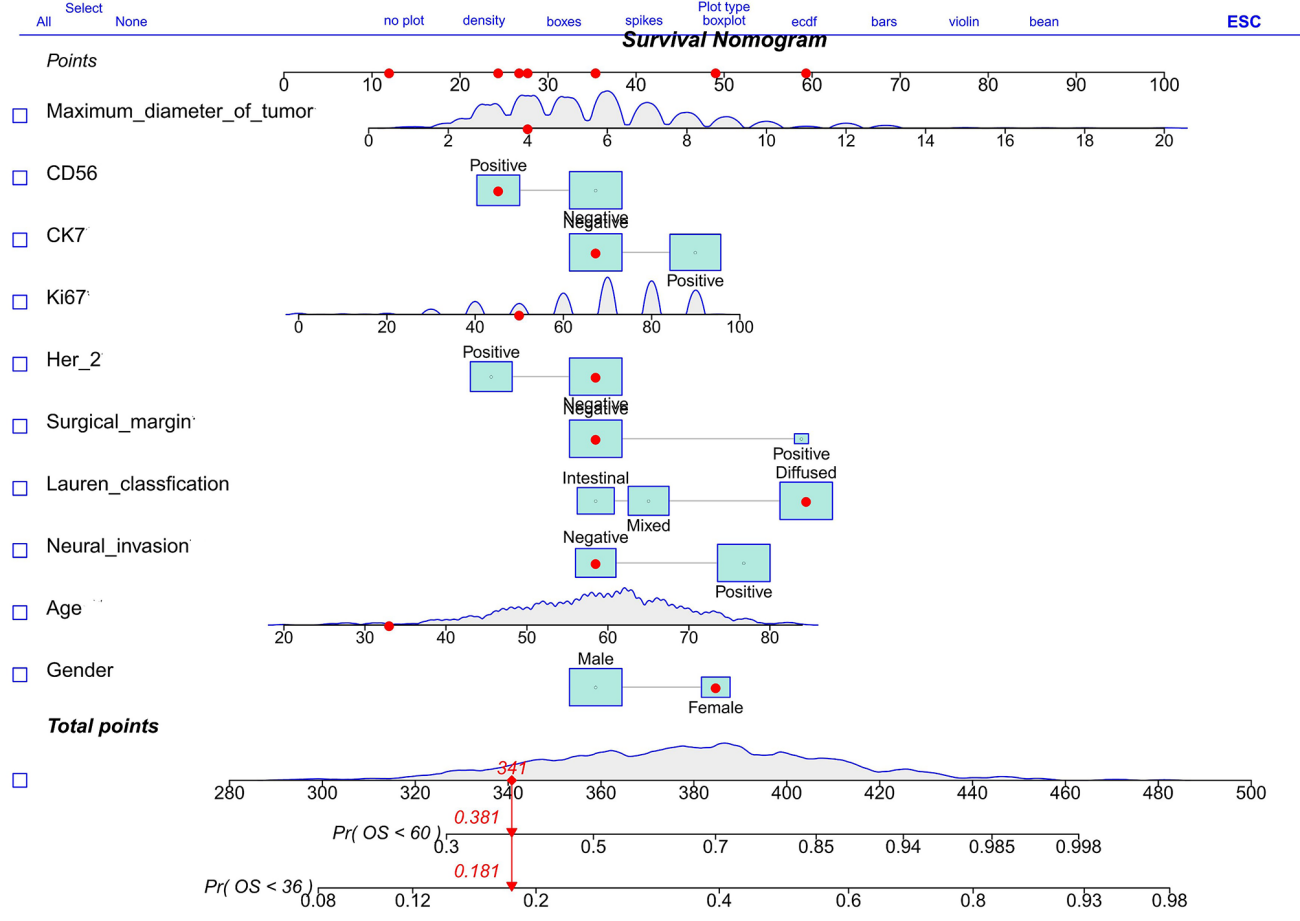
Nomogram model designed to predict the 3-year and 5-year OS of patients with positive lymphatic metastasis

Table 3 presents the results of the multivariate Cox regression analysis on a training cohort of 778 patients with positive lymphatic metastasis in a nature-inspired style. The analysis identifies independent risk factors that impact OS, including gender, age, maximum tumor diameter, neural invasion, Lauren classification, and expression of Her-2, CK7, and CD56. These nine variables are utilized to create a nomogram for estimating the 3-year and 5-year OS in gastric cancer patients with positive lymphatic metastasis. The nomogram serves as a valuable tool for identifying patients who are likely to have positive outcomes. Figure 8 showcases the nomogram model, incorporating the independent predictors mentioned above, to predict the 3-year and 5-year OS. In the training cohort, the C-index for predicting OS is 0.674, with a 95% confidence interval of 0.646–0.702. When compared to the discriminatory ability of the AJCC 8th edition TNM staging, the nomogram demonstrates superior performance with a higher C-index of 0.595 and a 95% confidence interval of 0.575–0.615.

**Table 3:** Multivariate analysis of OS of training cohort of positive lymphatic metastasis and analyzed by Cox regression

	B	SE	Wald	df	*p*	Exp(B)	EXP(95%CI)
Gender				1			
Male Vs. Female	0.282	0.120	5.504	1	0.019	1.326	1.048–1.679
age	0.020	0.005	14.022	1	< 0.001	1.020	1.010–1.031
Neural invasion				1			
Negative Vs. Positive	0.244	0.119	4.227	1	0.040	1.276	1.011–1.610
Lauren classification			8.643	2	0.013		
Intestinal Vs. Diffuse	0.387	0.148	6.858	1	0.009	1.473	1.102–1.968
Intestinal Vs. Mixed	0.103	0.162	0.407	1	0.524	1.109	0.807–1.522
Surgical margin				1			
Negative Vs. Positive	0.437	0.185	5.583	1	0.018	1.547	1.077–2.223
Her-2				1			
Negative Vs. Positive	-0.248	0.106	5.445	1	0.020	0.780	0.633–0.961
Ki67	0.009	0.004	6.244	1	0.012	1.009	1.002–1.017
CK7				1			
Negative Vs. Positive	0.351	0.123	8.173	1	0.004	1.420	1.117–1.806
CD56				1			
Negative Vs. Positive	-0.351	0.133	6.912	1	0.009	0.704	0.542–0.915
Maximum diameter of Tumor	0.072	0.020	12.557	1	< 0.001	1.075	1.033–1.118

Abbreviations: B: regression coefficient; SE: standard error; *df*: degree of freedom; HR: hazard ratio; CI: confidence interval

**Fig. 9:**
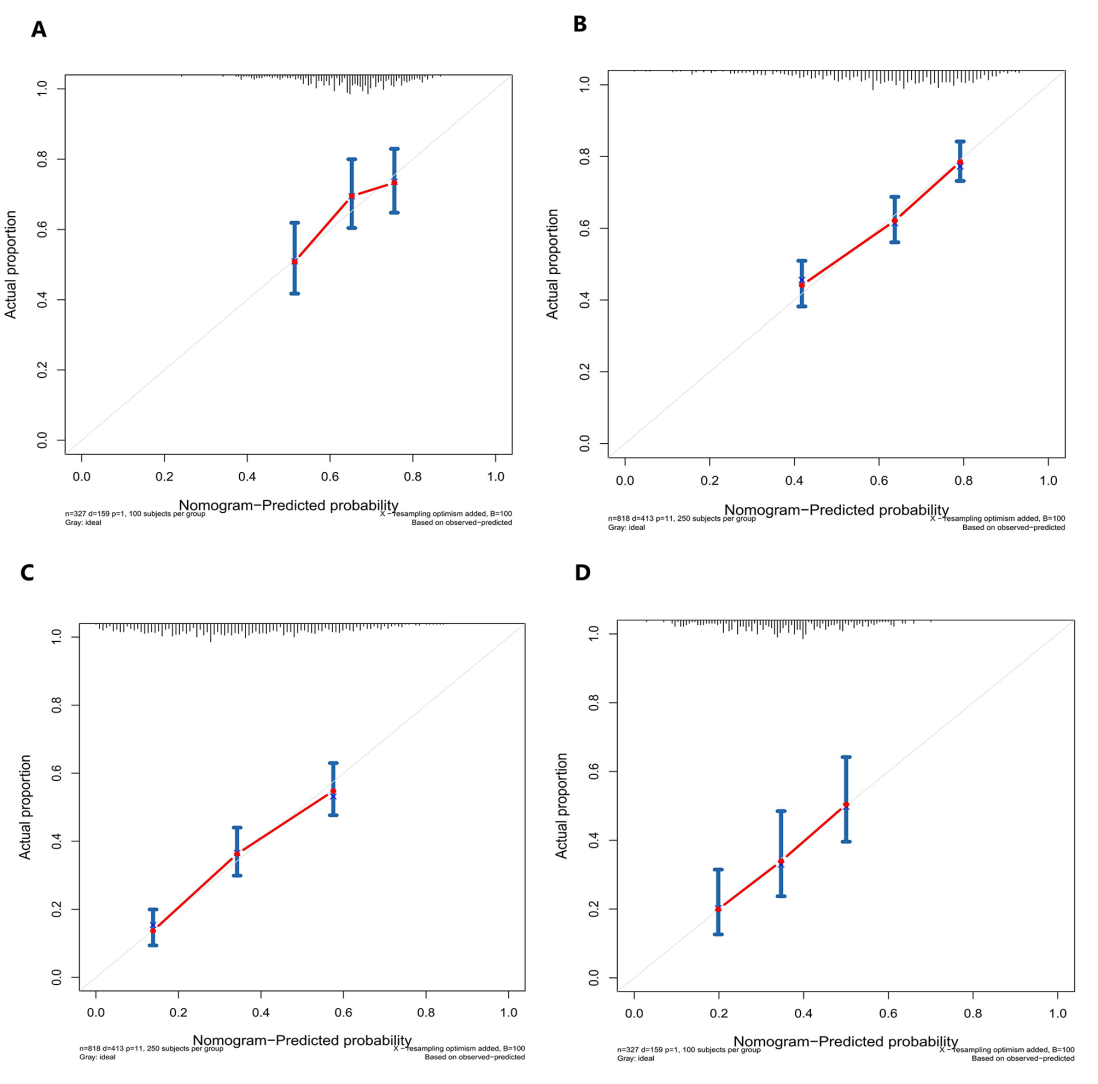
Calibration curves illustrating the accuracy of the internal and external validation in predicting the 3-year and 5-year OS of patients with positive lymphatic metastasis. **A**. Internal validation of 3-year OS. **B**. External validation of 3-year OS. **C**. Internal validation of 5-year OS. **D**. External validation of 5-year OS

**Fig. 10:**
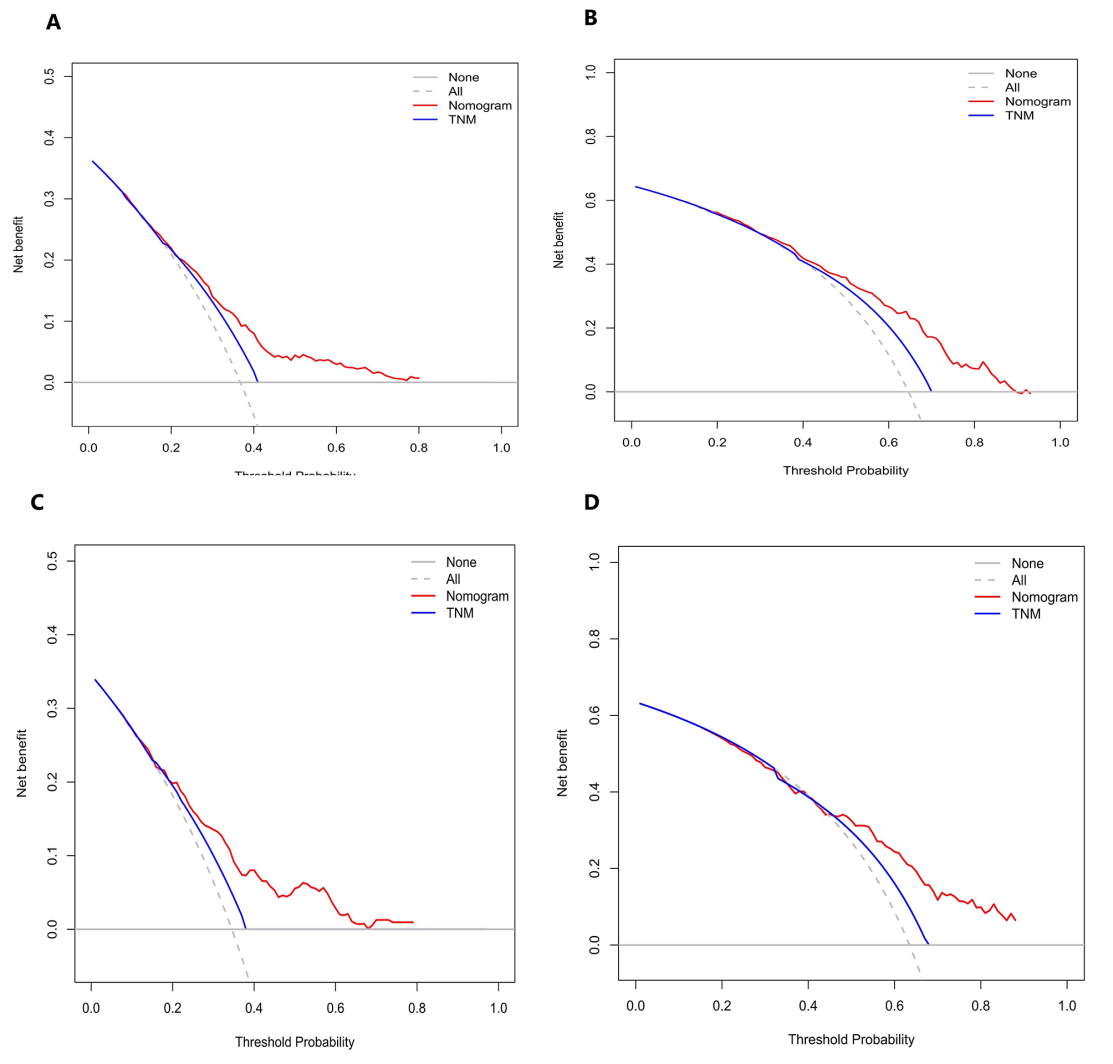
Decision curve analysis (DCA) results of the internal and external validation to predict the 3-year and 5-year OS of patients with positive lymphatic metastasis. **A**. Internal validation of 3-year OS. **B**. External validation of 3-year OS. **C**. Internal validation of 5-year OS. **D**. External validation of 5-year OS

The results presented in Fig. 9A, B and C, and 9D demonstrate the excellent calibration of the nomogram’s predictions, with both internal and external validations closely aligning with actual observations. Further assessment of the nomogram’s accuracy through time-dependent receiver operating characteristic (t-ROC) curve analysis revealed impressive area under the curve (AUC) values for the 3-year and 5-year overall survival (OS) models. The internal validation AUC for the 3-year OS was 0.702 (95%CI: 0.668–0.751), while the external validation AUC was 0.617 (95%CI: 0.548–0.694). Similarly, for the 5-year OS, the internal validation AUC was 0.752 (95%CI: 0.726–0.824), and the external validation AUC was 0.673 (95%CI: 0.537–0.724), exceeding expectations and highlighting the model’s exceptional performance (see Fig. 4C and D). Decision analysis curves (DCA) depicted in Fig. 10A, B and C, and 10D further underscore the clinical benefits of our nomogram, showcasing its superiority over the AJCC TNM classification. In both the training and validation cohorts, the nomogram yielded a higher net benefit compared to the AJCC TNM staging system. The internal validation C-index of 0.674 (95%CI: 0.646–0.702) outperformed the C-index of the AJCC 8th edition TNM staging, which stood at 0.595 (95%CI: 0.575–0.615). External validation supported these findings, with a C-index of 0.63 (95%CI: 0.581–0.68) for our nomogram compared to the AJCC 8th edition TNM staging’s C-index of 0.566 (95%CI: 0.535–0.598). Overall, these results validate the robustness and effectiveness of our nomogram in predicting outcomes and guiding clinical decision-making.

### Risk scoring of stratification system of OS for positive lymphatic metastasis

Based on the final nomogram model, each patient’s score was calculated, and a cutoff value for overall survival (OS) was established using the X-tile software in the training cohort of 778 patients. The log-rank test was then performed to compare survival times among different risk groups. The prognostic nomogram was used to calculate the total scores, and a cutoff value of 173.091 was utilized to divide the entire cohort of 1093 patients into two groups with distinct probabilities of disease progression risk (Fig. 6D, E and F). The low-risk group (0 ≤ 173.091) comprised 380 patients from the training cohort and 157 patients from the validation cohort. On the other hand, the high-risk group (> 173.091) contained 398 patients from the training cohort and 158 patients from the validation cohort. Figure 6 demonstrates the overall survival curves stratified by risk scores for all cohorts, with *p*-values less than 0.001 for each cohort. The median overall survival of the low-risk group in the entire cohort, training cohort, and validation cohort was 70, 72, and 58 months, respectively. In contrast, the median overall survival of the high-risk group in the three cohorts was 38, 37, and 40 months, respectively. These results indicate that our model effectively stratifies patients based on their risk and demonstrates good prognostic performance. Figure 7B illustrates the correlation between risk score and overall survival rate, showing a clear decline in the 5-year survival rate as the risk score increases. This trend is particularly evident when the score reaches 100 or higher, with survival rates dropping to 61.2% in the 100–130 range, 9.1% in the 220–250 range, and 19.3% in the 250–303 range. Similarly, the 3-year survival rate experiences a significant decrease when the risk score surpasses 130, decreasing from 86.7% in the 130–160 range to 19.3% in the 250–303 range. These findings provide a visual representation of the relationship between risk score and survival outcomes, aligning with the risk scoring of the OS stratification system. This underscores the inherent connection between risk assessment and survival prediction in this study.

## Discussion

Accurate lymph node staging plays a pivotal role in determining the most effective treatment strategy and predicting treatment outcomes for patients diagnosed with gastric cancer. This aspect holds significant importance, as lymph node metastasis serves as a crucial prognostic factor. There has been substantial scholarly research investigating the intricate connection between lymph node metastasis and the prognosis of gastric carcinoma [14–17]. The precise determination of lymph node staging is affected by both the anatomical location of metastatic lymph node metastasis and the extent of lymph node dissections [18, 19]. The expansion of lymph node dissection can gradually reduce or even prevent the migration of the lymph nodes stage, contributing to improved outcomes [20, 21]. The notion of lymph node ratios was introduced to encompass not only the number of metastatic lymph nodes but also the extent of lymphadenectomy, thus accounting for both aspects. Marchet A. et al. initially proposed a classification for gastric cancer based on the ratio of positive lymph nodes to the number of nodes examined [22]. In a retrospective analysis of 804 patients who underwent surgical resection for gastric cancer, Spolverato G. et al found that lymph node ratios were the most effective way to categorize patients based on lymph node status [14]. Kong SH. et al demonstrated that lymph node ratios system can account for the stage migration effect and effectively differentiate between lymph node stages when a sufficient number of nodes are examined [23]. It is worth noting that the specific boundary value for lymph node ratios may vary in different studies.

Our investigative study delved into the realm of gastric cancer, focusing on a group of patients diagnosed with this challenging disease. With meticulous attention to detail, we delved deep into their prognostic data over an extended period of time, seeking to uncover meaningful insights that could shape the way we approach treatment and care for these individuals. Utilizing a blend of COX regression and a variety of clinical, pathological, and molecular markers, we crafted a forest plot that serves as a powerful tool in predicting the overall survival of patients with and without lymph node metastasis. This intricate plot encompassed a range of variables, from gender and age to tumor characteristics and molecular expressions, painting a comprehensive picture of factors that could influence patient outcomes. In our quest for accuracy and reliability, we underwent rigorous internal and external validations, ensuring that our model stood up to the test of different medical settings and patient populations. The results of these validations were highly encouraging, affirming the strength of our predictive model in terms of accuracy, calibration, discrimination, and overall clinical utility. Going beyond mere validation, we delved into a population-based analysis, carving out distinct risk groups that further refined the predictive power of our forest plot. By segmenting patients based on their unique risk profiles, we empowered clinicians to make more informed and personalized treatment decisions, ultimately enhancing the quality of care delivered to those battling gastric cancer. Our holistic approach not only benefits patients by offering a clearer roadmap for their treatment journey but also equips physicians with a valuable tool to aid in their decision-making process. By marrying the complexities of gastric cancer with the precision of predictive modeling, our study aims to elevate the standard of care for all individuals affected by this challenging disease.

The TNM staging system is currently the primary tool used for predicting the risk of cancer in clinical practice. However, its accuracy and reliability are limited, which reduces its effectiveness [24–26]. Studies have shown that incorporating column charts can improve the accuracy of assessment and reduce unnecessary examinations for patients with gastric cancer [27]. Numerous scientific studies have explored prognostic factors for gastric cancer, including age, gender, tumor size, number of positive lymph nodes, depth of invasion, tumor location, Lauren classification, histological classification, and biomarkers. As a result, several prognostic models have been developed [28–34].

We have designed a prognostic score plot with a nature-inspired aesthetic to create a predictive and risk stratification model. This model accurately distinguishes between patients with and without lymphatic metastasis, allowing for personalized treatment plans. By identifying low-risk patients who may not require further therapy and high-risk patients who could benefit from targeted treatments, our model offers valuable insight for effective medical decision-making.

Nevertheless, it is important to highlight the limitations of our current research. Firstly, the model was only developed and validated using data from a single medical center, which may impact the generalizability of the results. Therefore, it is necessary to replicate these findings in other medical centers to ensure the reliability of the model. Moreover, there appears to be a discrepancy between the predictive ability of the line plot model for 5-year overall survival (OS) and the actual data from the patient groups, indicating the need for further investigation to understand this inconsistency. Additionally, our study did not distinguish between early and late-stage gastric carcinoma patients, which could affect the accuracy of the model’s predictions for each stage. Future studies should consider stratifying patients based on disease stage to assess the model’s performance more accurately.

## Conclusion

This study provides valuable risk stratification models for lymphatic metastasis in gastric carcinoma, encompassing both node-positive and negative cases. These models can help identify low-risk individuals who may not require further intervention, while high-risk individuals can benefit from targeted therapies aimed at addressing lymphatic metastasis.

## Data Availability

No datasets were generated or analysed during the current study.

## Declarations

### Registration of research

We registered our study on the Research Registry and the identifying number is Research Registry 9867 (https://researchregistry.knack.com/research-registry#user-researchregistry/).

## Abbreviations

AE1/AE3: Pan Cytokeratin Monoclonal Antibody
CD 56: Cluster of Differentiation 56
CDX-2: Caudal-related homeobox transcription factor 2
CGA: Chromogranin A
CK20: Cytokeratin 20
CK7: Cytokeratin 7
MLH 1: MutL homologous gene 1
MSH2: MutS homolog 2
MSH6: MutS homolog 6
OS: Overall survival
PFS: Progression-free survival
PMS 2: Postmitotic segregation increased 2
STAB-2: Stabilin-2
SYN: Synaptophysin
